# Supplementary figures and images for: Tiberius: end-to-end deep learning with an HMM for gene prediction
Source: Bioinformatics. 2024 Nov 18;40(12):btae685. doi: 10.1093/bioinformatics/btae685 (PMC11645249; doi:10.1093/bioinformatics/btae685)

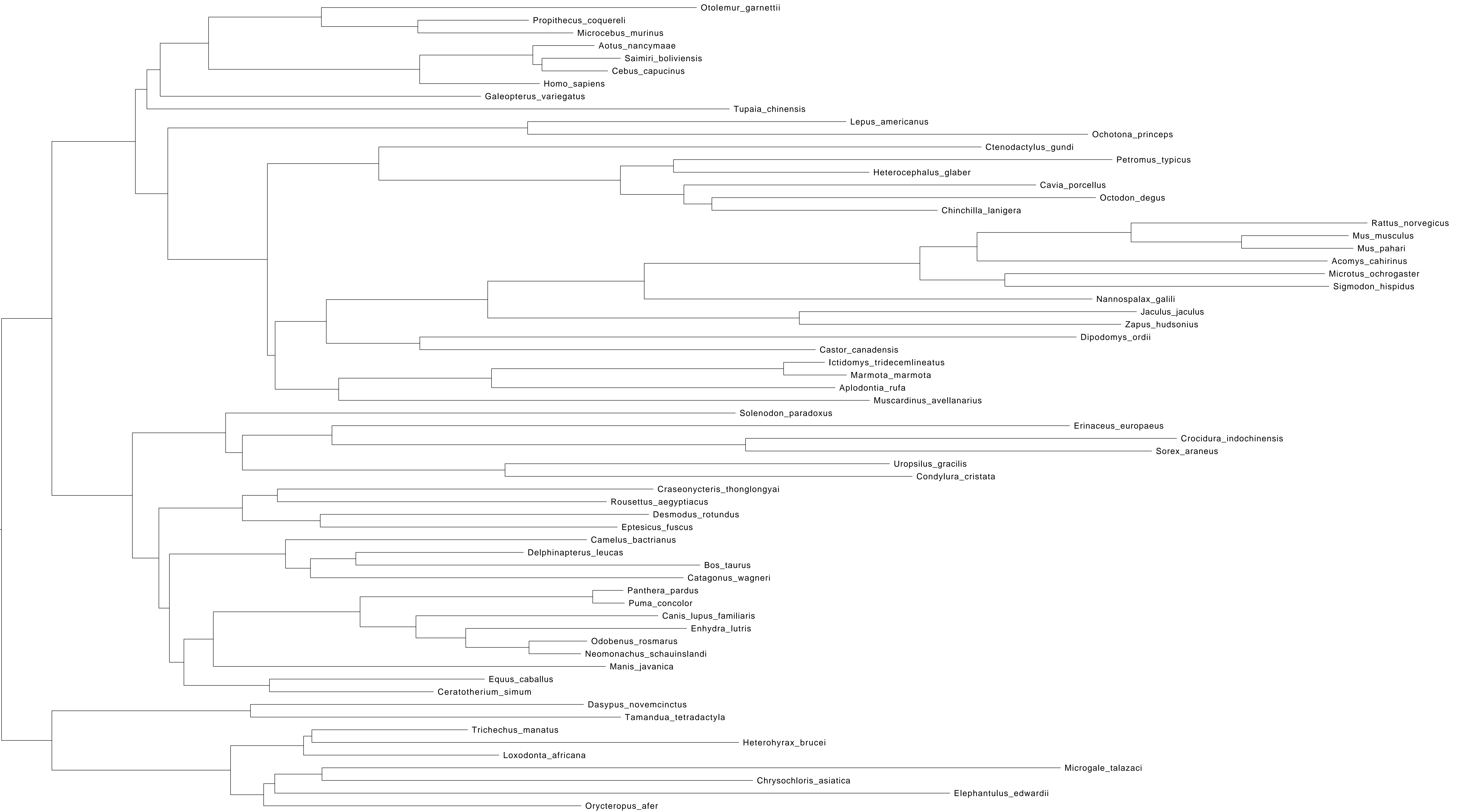

Supplement: btae685_Supplementary_Data [file btae685_supplementary_data.zip › assorted64.pdf]
